# Development of an Aryloxazole Derivative as a Brain-Permeable Anti-Glioblastoma Agent

**DOI:** 10.3390/pharmaceutics11100497

**Published:** 2019-09-28

**Authors:** Seulgi Shin, Sungsu Lim, Ji Yeon Song, Dohee Kim, Min Jeong Choi, Changdev G. Gadhe, A Young Park, Ae Nim Pae, Yun Kyung Kim

**Affiliations:** 1 Convergence Research Center for Diagnosis, Treatment and Care System of Dementia, Brain science institute, Korea Institute of Science and Technology (KIST), Seoul 02791, Korea; seulgishin@kist.re.kr (S.S.); sungsulim@kist.re.kr (S.L.); aster1214@kist.re.kr (J.Y.S.); 213015@kist.re.kr (D.K.); mjchoi@kist.re.kr (M.J.C.); gadhe.changdev@gmail.com (C.G.G.); 114054@kist.re.kr (A.Y.P.); 2 Department of Biological Chemistry, University of Science and Technology (UST), Daejeon 34113, Korea

**Keywords:** aryloxazole derivative, P-glycoprotein inhibition, anti-glioblastoma agent

## Abstract

Glioblastoma drug development has been difficult due to the extremely low blood brain barrier (BBB) penetration of conventional anti-cancer agents. P-glycoprotein, an efflux membrane transporter, is responsible for the poor brain uptake of small and hydrophobic drug substances. To develop brain-penetrable anti-tumor agents, we designed colchicine derivatives containing an aryloxazole moiety, which is known to inhibit P-glycoprotein. Among those tested, an aryloxazole derivative named KIST-G1 showed the strongest anti-glioblastoma cell proliferation activity (IC_50_ = 3.2 ± 0.8 nM). Compared to colchicine, KIST-G1 showed dramatically increased BBB-permeable properties presenting 51.7 ± 0.5 (10^−6^ cm/s) parallel artificial membrane permeability assay (PAMPA) permeability and 45.0 ± 6.0% of P-gp inhibition. Aid by the BBB-permeable properties, KIST-G1 (5 mg/kg) suppressed glioblastoma cell growth and migration almost completely in the brain of glioblastoma xenograft models by showing 98.2 ± 0.1% reduced tumor area compared with phosphate buffered saline (PBS)-injected control. In comparison, temozolomide, which is the most widely used drug for glioblastoma, showed only moderate effects. Our results demonstrate the effectiveness of an aryloxazole moiety in targeting brain tumors and suggest KIST-G1 as a potent anti-glioblastoma agent.

## 1. Introduction

Glioma is a collection of tumors arising from glial cells. Clinically, the most malignant type of glioma is classified as glioblastoma (GBM) [1]. The current standard treatment for GBM is surgical resection combined with chemo- and radiation therapy [2]. Despite the aggressive treatment, most patients develop recurrent glioblastoma, and the median survival time of the patients is merely six months after the recurrence [3,4]. To increase the survival rate, more effective anti-glioblastoma agents that suppress tumor growth and migration are urgently required. 

The major difficulty in drug development for GBM comes from the low blood brain barrier (BBB) penetration of conventional anti-cancer drugs. The BBB is a physical membrane barrier that separates the circulating blood from the brain extracellular fluid. Small and hydrophobic drug substances are generally expected to penetrate the BBB. However, in reality, a number of drug substances exhibit poor brain uptake profiles due to P-glycoprotein (P-gp) efflux [5,6]. Accumulating data suggest that P-gp inhibition by verapamil increases brain uptake of conventional anti-cancer drugs such as paclitaxel and colchicine [7,8]. Therefore, we envisioned that the design of a new drug scaffold based on arylthiazole or aryloxazole moieties, of which structures are known to inhibit P-gp [9], would be beneficial in targeting brain tumors. 

Toward that, we developed a series of arylthiazole or aryloxazole derivatives, which are designed to fit in the colchicine binding site of tubulin. Colchicine is one of the representative anti-microtubule agents, which have potential anti-tumor effects [10]. Despite its BBB-penetrable size and hydrophobicity, the brain uptake rate of colchicine is extremely low due to the P-gp efflux [11]. Aryloxazole and arythiazole moieties are known to inhibit P-gp [9,12]. Therefore, we envisioned that the addition of an aryloxazole or an arythiazole moiety to colchicine would increase the BBB penetration and consequently increase the efficacy of the drug against glioblastoma. Previously, we demonstrated the anti-microtubule and the anti-mitotic activities of 80 aryloxazole and arylthiazole derivatives [13]. In this study, we characterized the anti-glioblastoma activities of the derivatives and the present compound, KIST-G1, as a potential anti-glioblastoma agent targeting the brain.

## 2. Materials and Methods 

### 2.1. Glioblastoma Cell Culture 

Human glioblastoma cell lines (A172, U251, U373, T98G, HS683, and U87MG) were purchased from American Type Culture Collection (ATCC) (Manassas, VA, USA). Glioblastoma cells were grown in Dulbecco’s Modified Eagle Medium (DMEM) containing 10% fetal bovine serum (FBS), 100 units/mL penicillin, and 100 μg/mL streptomycin at 37 °C in a humidified atmosphere containing 5% CO_2_.

### 2.2. Primary Screening of Small Molecules 

From an in-house library composed of small molecules targeting the colchicine binding site of P-gp, 80 aryloxazole and arylthiazole derivatives were tested for anti-proliferative effects on glioblastoma cells. For primary screening, U87MG cells were seeded in 384-well plates using DMEM with 10% FBS and then treated with 10 μM of each candidate compound for 24 h [14]. After 24 h, nuclei were counterstained with 2 µg/mL Hoechst (Invitrogen, Waltham, MA, USA). The anti-proliferative effects of the candidate compounds were determined by comparing the number of cells in the compound-treated group with the number of cells in the control group.

### 2.3. Glioblastoma Cell Proliferation Assay 

For anti-proliferative activity against various glioblastoma cells, A172, U251, U373, T98G, HS683, and U87MG cells were seeded into 96-well plates at a density of 0.5 × 10^4^ cells/well [15]. After 24 h of cell attachment, glioblastoma cells were treated with seven aryloxazole derivatives from in-house library (Scheme S1), colchicine (Sigma, St. Louis, MO, USA) (0.1 nM~10,000 nM), or temozolomide (Sigma) (0.01 mM~10 mM). After 48 h of the treatment, the cells were stained with calcein-AM (Invitrogen) (0.5 µM) and Hoechst (2 µg/mL) to determine cell viability and proliferation. The plates were then incubated at 37 °C for 30 min. The fluorescence response was imaged by using Operetta® (PerkinElmer™, Waltham, MA, USA) and analyzed by using Harmony 3.1 software (PerkinElmer™). Error bars indicate S.D. of triplicate experiments. The IC_50_ value was determined by Prism’s nonlinear regression analysis.

### 2.4. Glioblastoma Cell Migration Assay 

For glioblastoma cell migration assay, U87MG cells were seeded into 12-well plates at a density of 4 × 10^4^ cells/well. After 12 h of cell attachment, the monolayer of U87MG cells was scratched with a pipette tip across the center of the well [16]. The cells were washed twice with phosphate buffered saline (PBS) to remove cell debris and then were treated with temozolomide (0.01~10 mM), KIST-G1, or colchicine (0.1~100 nM) for 24 h. Cells were fixed with ice-cold 100% methanol and then stained with 0.06% trypan blue solution. The number of migrated U87MG cells into an empty space (0.26 mm^2^) were counted by using Image J software (NIH) (Bethesda, MD, USA). Error bars represent the S.D. of triplicate experiments. Quantification data were analyzed by Student’s *t*-test.

### 2.5. Immunofluorescence Stain 

For microtubule staining of glioblastoma cells, U87MG cells were seeded at 0.4 × 10^4^ cells/well in a 96-well plate and incubated 24 h prior to the addition of compounds. Temozolomide (0.01~10 mM), colchicine, or KIST-G1 (0.1~100 nM) were treated to cells for 48 h. After the compound treatment, U87MG cells were fixed in 3.7% formaldehyde, permeabilized, and stained using standard procedures. Anti-beta III tubulin antibody (AbCam, Cambridge, MA, USA) was used as a primary antibody at a 1:500 dilution and incubated overnight at 4 °C. Alexa-488 conjugated phalloidin (Sigma) was used to visualize filamentous actin.

### 2.6. Generation of Subcutaneous Glioblastoma Xenograft Model and Drug Treatment

Subcutaneous glioblastoma-bearing mice were obtained by a subcutaneous injection of 1 × 10^7^ U87MG cells within 100 μL DMEM into the flanks of 14-week-old female BALB/c nude mice [17]. Then, 17 days after tumor cell implantation, when the average tumor size reached approximately 700 mm^3^, mice were randomly divided into four groups with seven mice per group. Animals were treated intraperitoneally with a vehicle, KIST-G1 (1 mg/kg or 10 mg/kg), or temozolomide (TMZ) (10 mg/kg) in 100 µL of PBS containing 5% DMSO. A total of seven administrations were performed every other day throughout the two week duration. Body weight and tumor size of each mouse were also measured every other day during the two weeks. Tumors were measured with a digital caliper and estimated using the following formula: *Tumor volume = (length × width^2^)/2*. All mice were sacrificed after the tumor size of the PBS-treated group reached 3000 mm^3^. Tumor tissues were surgically excised, fixed by 4% paraformaldehyde solution, and frozen in compound (Leica, Wetzlar, Germany).

For all animal experiments, mice were housed on a 12:12 h light-dark cycle in pathogen-free facilities at the Korea Institute of Science and Technology. Animal protocols followed the principles and practices outlined in the approved guidelines by the Institutional Animal Care and Use Committee of the Korea Institute of Science and Technology. All animal experiments were approved by the Korea Institute of Science and Technology (KIST-2019-032; 1 April 2019).

### 2.7. Generation of Orthotopic Glioblastoma Xenograft Model and Drug Treatment

Orthotopic glioblastoma-bearing mice were obtained by an intracranial implantation (coordinate; AP +0.5, ML +1.7, DV −3.2 mm from Bregma) of 1 × 10^5^ U87MG cells within 5 μL PBS into the brain of 8-week-old male BALB/c nude mice [18]. Three days after tumor cell implantation, mice were randomly divided into three therapeutic groups with four mice per group. Mice in each group were injected intraperitoneally with a vehicle, 5 mg/kg of KIST-G1, or 5 mg/kg of TMZ [19] in 100 μL PBS containing 5% DMSO. Twelve total administrations were performed three times a week during 28 days. All therapeutic groups were sacrificed 28 days after initial treatment. Brains were removed, fixed by 4% paraformaldehyde solution, and frozen in OCT compound (Leica, Wetzler, Germany).

### 2.8. Immunohistochemical and Immunofluorescence Analysis 

For immunohistochemical analysis, tumor tissues of 10* *µm thickness from a subcutaneous glioblastoma xenograft model were stained with hematoxylin (Gill No.1, Sigma) and eosin (5 wt % in H_2_O, Sigma). To estimate tumor volumes, tissue slices of 40 μm thickness from the brain of orthotopic glioblastoma xenograft models were stained with cresyl violet (Sigma) [20]. Tumor areas (AP +1.0 and AP −3.0 mm) were measured by ImageJ software. 

For the immunofluorescence staining procedure, tissue sections of 40 µm thickness from a subcutaneous glioblastoma xenograft model were stained with anti-cleaved caspase-3 (1:200; Cell Signaling Technology, Danvers, MA, USA) antibody. Immunofluorescent staining was performed with anti-CD44 (1:200, Cell Signaling Technology) antibody to evaluate the migration of glioblastoma cells into the contralateral hemisphere. Nuclei were counterstained with Hoechst. Images were acquired using a Nikon Eclipse inverted microscope (T*i*, Nikon, Tokyo, Japan).

## 3. Results

### 3.1. Anti-Glioblastoma Activities In Vitro

**Figure 1:**
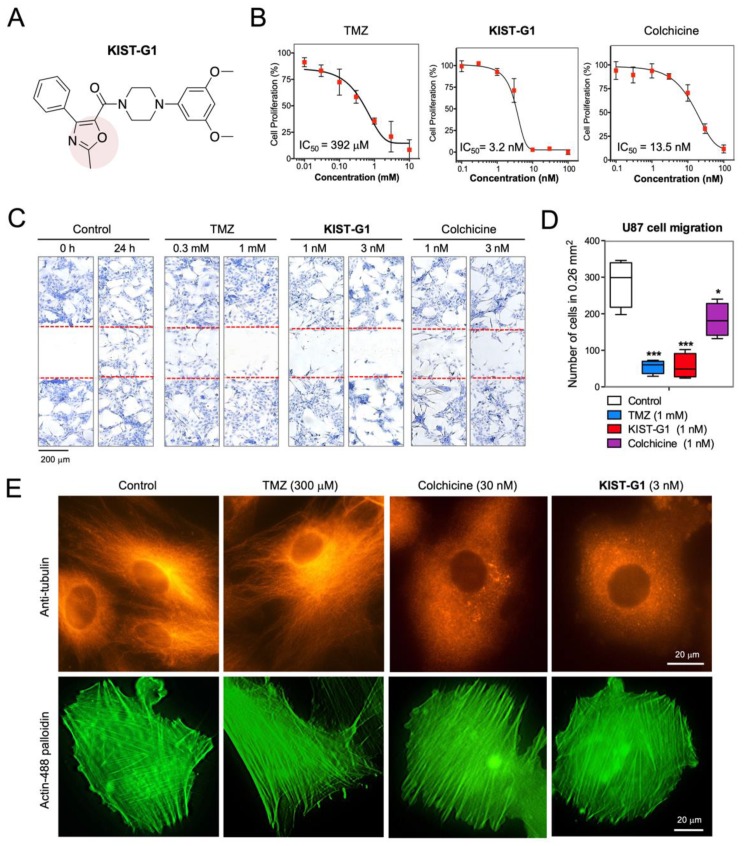
Anti-proliferating and anti-migration activity of KIST-G1. (**A**) Chemical structure of KIST-G1. (**B**) U87MG cells were plated on 96-wells and then treated with temozolomide (TMZ), KIST-G1, or colchicine at different concentrations for 48 h. The inhibitory effects of TMZ, KIST-G1, or colchicine on U87MG cell proliferation were evaluated by staining with Hoechst nuclei dye. Error bars represent the standard deviations of three independent experiments. IC_50_ value was determined by nonlinear regression analysis employing GraphPad Prism. (**C**) Monolayer of U87MG cells was scratched with a pipette tip and treated with TMZ, KIST-G1, or colchicine at indicated concentrations. After 24 h, cells were fixed with methanol and then stained with 0.06% trypan blue solution. Scale bar, 200 µm. (**D**) Number of migrated U87MG cells into an empty space (0.26 mm^2^) were counted by using Image J software. Error bars represent the standard deviations. *** *p* < 0.001, * *p* < 0.05. (**E**) U87MG cells were treated with TMZ (300 µM), colchicine (30 nM), or KIST-G1 (3 nM). After 48 h, cells were fixed and stained with anti-betaIII tubulin antibody and fluorescent-labeled phalloidin. Scale bar, 20 µm.

To evaluate anti-glioblastoma activity, we screened 80 aryloxazole and arylthiazole derivatives against glioblastoma-cell proliferation. For the primary screening, we used U87MG, which is the most well-characterized grade IV glioma cell line. Grade IV glioma is the most common primary brain tumor, called glioblastoma multiform (GBM) [21]. For cell proliferation assay, U87MG cells plated on 384-well plates were treated with each compound at 10 µM. After 24 h, nuclei were stained with Hoechst for 1 h. The Hoechst images were acquired by using Operetta®, and the nuclei were counted by using Harmony 3.1 software. As a result, seven aryloxazole derivatives showed strong anti-glioblastoma cell proliferation activity. To select the most effective compound against various GBM cell types, the anti-glioblastoma activity was further evaluated against six glioblastoma cell lines, A172, U251, U373, T98G, U87MG, and HS683 (Table S1). Among the selected seven compounds, the compound with 3,5-dimethoxy phenyl piperazine (KIST-G1) showed better anti-proliferative activities than 3-chloro, 3-methoxy, and 3,5-dichloro substituted phenyl moiety (KIST-G2 and KIST-G3) by comparing activities of compound KIST-G1. It seemed that electron donating (methoxy) was preferable to the electron-withdrawing (chloro) groups for better activity. Regarding oxazole substituents, the compound KIST-G1 without substituent was better than 2, 3, or 4-fluorine substituents at oxazole moiety. It seemed 2 and 4-substituents were tolerated, but 3-fluoro substituent was more detrimental to the activity of compounds. K1ST-G1 showed the strongest anti-proliferation effect against all six glioblastoma cell lines at a nanomolar concentration (Figure 1A). The anti-proliferation activity against U87MG of KIST-G1 (IC_50_ = 3.2 ± 0.8 nM) was stronger than that of colchicine (IC_50_ = 13.5 ± 1.2 nM) (Figure 1B and Figure S1). In the case of temozolomide (TMZ), the most widely used drug to treat glioblastoma [22], the anti-proliferation activity was merely 392 ± 3.7 µM against U87MG cells.

Wound-healing assay was followed to evaluate the compound’s effect on glioblastoma cell migration. Monolayer cultures of U87MG cells were scratched with a pipette tip and incubated with each compound for 24 h. In the control wells, U87MG cells were migrated and filled up the wounded region (Figure 1C). In contrast, U87MG cell migration was almost completely inhibited by the treatment of KIST-G1 at 1 nM, a concentration lower than its half inhibitory concentration (3.2 ± 0.8 nM) against U87MG cell proliferation (Figure 1D). Compared with KIST-G1, colchicine treatment showed only 43.7 ± 7.9% inhibitory effect on U87MG cell migration. In the case of TMZ, U87MG cell migration was inhibited at 1 mM, a concentration higher than its half inhibitory concentration (392.1 ± 3.7 µM) against U87MG cell proliferation. The in vitro assays showed that KIST-G1, compared to colchicine, more effectively inhibited glioblastoma cell proliferation and migration.

Next, we further evaluated the anti-microtubule effect of KIST-G1 as an anti-microtubule agent mimicking colchicine. U87MG cells were treated with each compound for 48 h and then stained with anti-microtubule antibody. Figure 1E shows that well-organized microtubule structures were observed in control and TMZ-treated cells. In contrast, massive disruption of the microtubule network was observed in KIST-G1-treated cells as well as colchicine-treated cells (Figure 1E). In all of these cases, actin filaments were not affected, implying that KIST-G1 disrupted microtubule selectively and did not affect other macrostructures in the cell. The in vitro results indicate that KIST-G1 was a highly potent anti-microtubule agent presenting stronger anti-glioblastoma activity than colchicine.

### 3.2. AP-gp Inhibition by KIST-G1 

**Figure 2:**
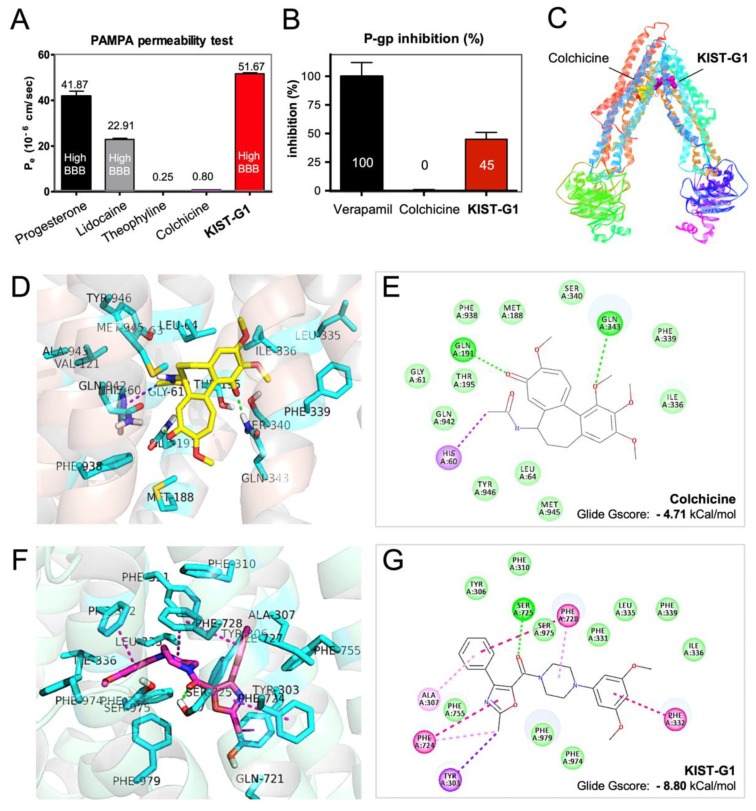
KIST-G1 as a blood brain barrier (BBB)-penetrable anti-microtubule agent. (**A**) Permeabilities of KIST-G1 and colchicine were determined by a parallel artificial membrane permeability assay (PAMPA)-BBB system. Permeability (P_e_) classification; high BBB (P_e_ > 10 × 10^−6^ cm/s), low BBB (Pe < 10 × 10^−6^ cm/s). (**B**) Efflux of P-gp substrate across the LLC-PK1-MDR1 cell monolayers was measured in the absence or the presence of verapamil, colchicine, or KIST-G1 for 1 h. Each bar represents the mean with the standard deviations from three repeats. (**C**) Drug-binding pocket of mouse P-gp with colchicine and KIST-G1 as seen in the X-ray crystal structure (PDB 4XWK). (**D**–**G**) Interaction of colchicine (yellow) or KIST-G1 (purple) with P-gp. Close-up view of colchicine (**D**) and KIST-G1 (**F**) with P-gp are shown in sticks and surrounding 4 Å residues in lines. Conventional H-bond, Pi–Pi stacking, and Pi–Sigma interactions are represented by green, magenta, and violet dash lines. Transparent hydrogen bond donor/acceptor surfaces are shown for residues surrounding colchicine (**E**) and KIST-G1 (**G**).

To estimate the BBB permeability, a parallel artificial membrane permeability assay (PAMPA) test was administered (Figure 2A, Table S2) [23,24]. Progesterone and lidocaine were used as a highly BBB-permeable control, and theophylline was used as a negative control [24]. As previously reported, colchicine did not penetrate PAMPA at all. In contrast, KIST-G1 displayed a dramatically higher membrane permeability [51.7 ± 0.5 (10^−6^ cm/s)] than those of progesterone or lidocaine. Next, P-gp inhibitory activity was determined. For the P-gp efflux assay, LLC-PK1-MDR1 cells expressing P-gp were seeded onto transwell filter membranes. In the absence or the presence of each compound, the movement of a P-gp substrate (quinidine) across the transwell membrane was measured for 1 h, and the efflux ratio was calculated (Note S3) [25]. Quinidine efflux was completely inhibited by the treatment of verapamil, a representative inhibitor of P-gp (Figure 2B, Table S3) [26]. In the presence of KIST-G1, quinidine efflux was significantly reduced by showing a 45.0 ± 6.0% decrease. In the case of colchicine, quinidine efflux was not affected at all. The results clearly suggest that KIST-G1, because it was designed to contain the aryloxazole moiety, was effective in P-gp inhibition.

To investigate the role of the aryloxazole moiety in P-gp inhibition, a molecular docking study was followed by using a P-gp crystal structure co-crystalized with BDE-100 (PDB, 4XWK) [27] (Figure 2C). As a well-known substrate of P-gp, colchicine was docked into the drug binding site of P-gp. In the binding pocket, colchicine was docked mainly with Van der Waals and hydrogen bond interactions resulting in −4.71 kcal/mol of Glide Gscore and −41.08 of Emodel score (Figure 2D,E). In contrast, KIST-G1 was docked strongly into the deep crevice of the inhibitor binding site of P-gp. The Glide Gscore and the Emodel scores for KIST-G1 were −8.80 kCal/mol and −63.49, respectively (Figure 2F,G). The oxazole ring of KIST-G1, in particular, provided strong Pi–sigma and Pi–Pi interactions with tyrosine (Tyr303) and phenylalanine (Phe724, Phe728) residues. Additionally, the 3,5-dimethoxyphenyl ring of KIST-G1 formed hydrophobic interactions with the surrounding residues (Phe331, Phe332, Leu335, Ile336, Phe339, and Phe974). Ser725 residue formed a hydrogen bond with the carbonyl oxygen of KIST-G1. The computational docking study supports the importance of the aryloxazole moiety of KIST-G1 in P-gp inhibition.

### 3.3. In Vivo Test in Subcutaneous U87MG-Xenograft Model

**Figure 3:**
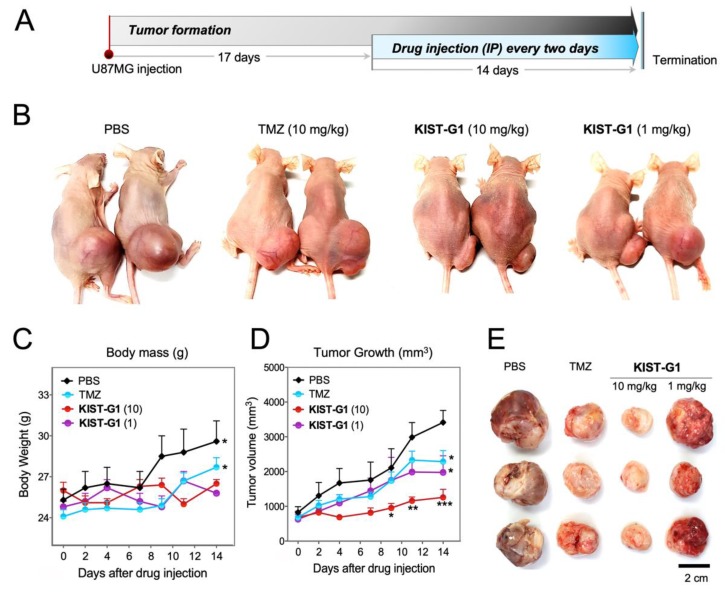
Anti-glioblastoma activity of KIST-G1 in a subcutaneous xenograft model. (**A**) U87MG cells were implanted into nude mice by subcutaneous injection of 1 × 10^7^ cells per animal. After 17 days of glioblastoma cell injection, U87MG tumor-bearing mice were treated intraperitoneally with phosphate buffered saline (PBS) (control), TMZ (10 mg/kg), or KIST-G1 (1 mg/kg or 10 mg/kg) every other day for 14 days (*n* = 7/group). (**B**) Representative images of subcutaneous U87MG tumor-bearing mice on the last day of drug administration. (**C**) Body weights and (**D**) tumor volumes of mice were measured every other day during drug administration. Values were individually expressed as the mean and the standard error of the mean, *n* = 7, * *p* < 0.05, ** *p* < 0.01 and *** *p* < 0.001. (**E**) Representative images of U87MG tumor tissues excised at the end of the experiment. Scale bar, 2 cm.

**Figure 4:**
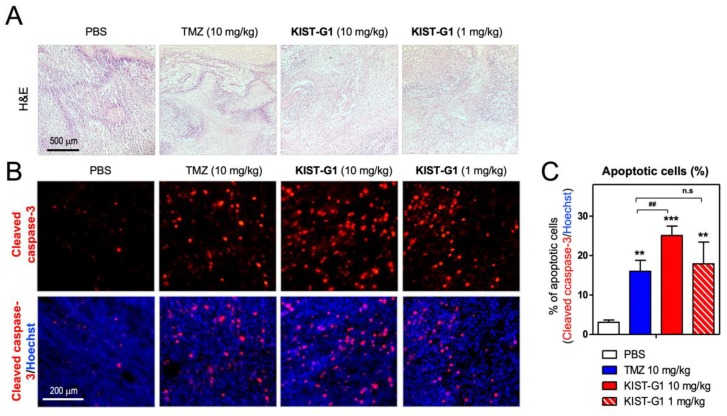
KIST-G1-induced apoptotic cell death in glioblastoma xenograft model. (**A**) Hematoxylin and eosin (H&E) stained images of tumor tissue sections from U87MG subcutaneous xenografts. Scale bar, 500 µm. (**B**) Immunofluorescent images with anti-cleaved caspase-3 (red). Blue indicates nuclei counter stained with Hoechst. Scale bar, 200 μm. (**C**) Percentage of apoptotic cells in histological tumor sections of U87MG subcutaneous xenografts. Data are expressed as the mean and the standard error of the mean, *n* = 3, ** *p* < 0.01 and *** *p* < 0.001.

To evaluate the anti-tumor effect of KIST-GI in vivo, U87MG cells were injected into mice subcutaneously. In the case of TMZ, 2.5~50 mg/kg doses were reported to be effective in both subcutaneous and orthotopic xenograft models [19,28,29,30]. To compare the effectiveness of KIST-G1 with TMZ in the subcutaneous model, we selected 1 and 10 mg/kg doses for KIST-G1 and 10 mg/kg for TMZ. After 17 days of tumorigenesis, mice (*n* = 7/group) were injected intraperitoneally (I.P.) with KIST-G1 (1 mg/kg or 10 mg/kg), TMZ (10 mg/kg), or PBS as a vehicle every other day for a period of 14 days (Figure 3A,B). Throughout the 14 days, PBS and TMZ-treated animals showed increasing body weights reflecting the growth of tumor volume, whereas KIST-G1-treated animals showed neither significant increases nor decreases in body weights (Figure 3C,D). In the subcutaneous xenograft model, the initial tumor volume was 826.7 ± 304.4 mm^3^. After two weeks of injection, PBS-treated animals displayed a tumor volume of 3415.3 ± 770.4 mm^3^ (Figure 3E), whereas KIST-G1-treated animals showed tumor volumes of 1975.1 ± 1068.5 mm^3^ at 1 mg/kg and 1257.7 ± 565.9 mm^3^ at 10 mg/kg. Considering the initial tumor volume preceding the drug injection, KIST-G1 inhibited tumor growth by 79.2% (10 mg/kg) and 51.8% (1 mg/kg) within two weeks. In contrast, TMZ injection inhibited tumor growth by merely 41.9% at 10 mg/kg treatment. Our findings indicate that KIST-G1 effectively inhibited tumor growth compared to TMZ. No anatomical changes of liver were observed in any of the animals, indicating that KIST-G1 did not cause liver toxicity (Figure S2). To scrutinize drug-induced toxicity further, body weights and blood creatinine levels were measured after the repeated injection of PBS, TMZ, or KIST-G1 to C57BL/6 mice for 28 days (Figure S3). In all cases, neither body weight nor creatinine levels were changed, indicating that there was no apparent sign of nephrotoxicity. Hematoxylin and eosin (H&E) stain displayed that PBS-treated group showed purple-colored stain, indicating high density of tumor cells in the tumor region (Figure 4A). In contrast, TMZ and KIST-G1-treated groups showed pink-colored stain, indicating comparably low density of tumor cells. Caspase-3 immuno-fluorescence stain further showed a number of apoptotic cells in KIST-G1-treated groups. In KIST-G1-treated groups (10 mg/kg), the rate of apoptotic cells was 25.1 ± 2.4% while the PBS-treated group showed only 3.1 ± 0.6% of apoptotic cells (Figure 4B,C). These results indicate the potential of KIST-G1 as an anti-tumor agent inducing apoptotic tumor cell death in subcutaneous U87MG xenograft models. To clarify the apoptotic pathway induced by KIST-G1, we investigated apoptotic markers such as cytochrome C and poly (ADP-ribose) polymerase (PARP) [31,32] (Figure S4). Upon the treatment of KIST-G1, apoptotic markers were significantly elevated by showing 1.6-fold increase in cytochrome C expression and 52.1 ± 3.5% increase in PARP cleavage. The results showed that KIST-G1 induced apoptotic cell death through the activation of caspase-dependent apoptosis.

### 3.4. In Vivo Test in Orthotopic U87MG-Xenograft Model

**Figure 5:**
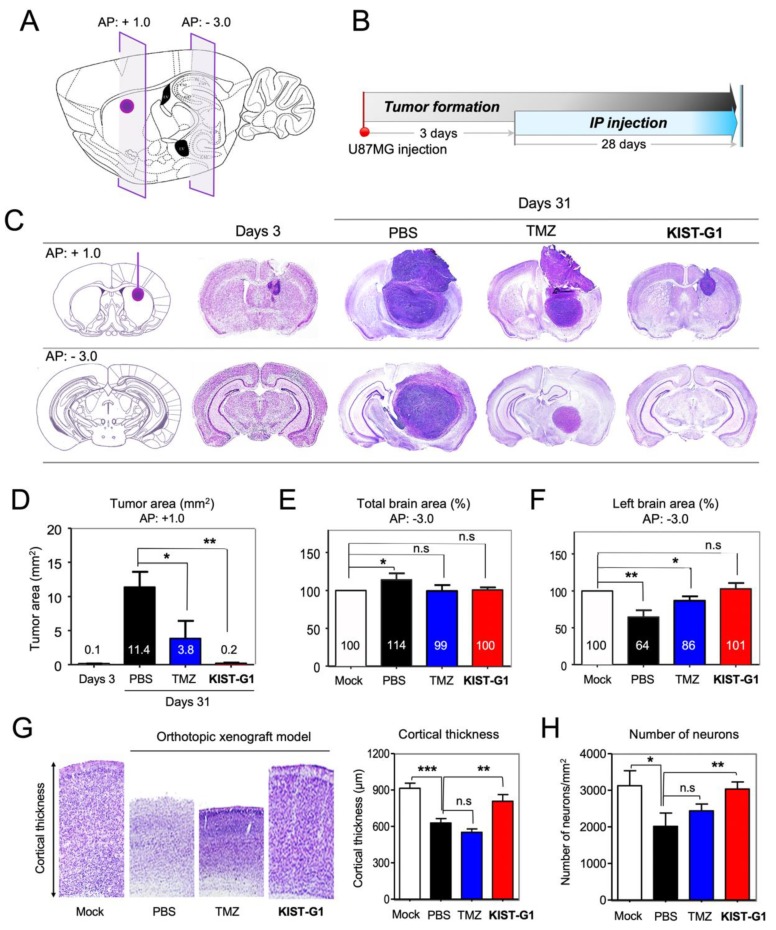
KIST-G1 reduced tumor volume in orthotopic xenograft mice brains. (**A**) The schematic diagram represents the generation of the orthotopic xenograft model by injecting U87MG cells into the striatum. (**B**) After three days of U87MG cell injection, orthotopic xenograft models of glioblastoma were treated with PBS, TMZ, or KIST-G1 (5 mg/kg) three times per week during 28 days (*n* = 4/group). (**C**) On day 3 and day 31, dissected sections of orthotopic xenograft mice brain were stained with cresyl violet to visualize tumor histology. (**D**) Tumor area (mm^2^), (**E**) total brain area (%), and (**F**) left brain area (%) of orthotopic xenograft mice brain were analyzed on the 31st day after U87MG glioblastoma cell injection. Data are expressed as the mean with a standard error, *n* = 4. (**G**) Magnified cortical areas of brain tissues in (C, AP: +1.0). Cortical thickness was measured using NIS-elements software (Nikon). The coronal section of a normal C57BL/6 mouse was used as a mock (control). Data are expressed as the mean and the standard error of the mean, *n* = 4. (**H**) Number of neurons in the cortex. Error bars indicate standard deviations from four animals in each group, *n* = 4, * *p* < 0.05, ** *p* < 0.01, *** *p* < 0.001 and n.s (not significant).

To confirm the anti-neoplastic effects of KIST-G1 for glioblastoma in the brain, U87MG cells were injected into the striatum of BALB/c nude mouse brain (AP: +1.0, ML: +1.7, DV: −3.2 mm). After three days of U87MG injection, the animals were intraperitoneally (I.P) treated with TMZ (5 mg/kg), KIST-G1 (5 mg/kg), or PBS for four weeks (*n* = 3) (Figure 5A,B). The mice were then sacrificed, and the brains were dissected for the histological analysis. Cresyl violet stain showed the histological features of glioblastoma tissues (Figure 5C). KIST-G1 (5 mg/kg) treatment dramatically decreased the growth of glioblastoma in the brain, indicating a 98.2 ± 0.1% reduced tumor area compared with PBS-treated control at the injection site (AP: +1.0 mm) (Figure 5D). The treatment of TMZ (5 mg/kg) also decreased glioblastoma by showing a 66.7 ± 22.6% reduced tumor area. In the case of PBS-injected control, the size of tumor at AP: −3.0 mm, which was far from the injection site, was as large as that of the injection site, indicating massive tumor growth and migration. In contrast, the KIST-G1-treated group showed no trace of tumor at AP: −3.0 mm, demonstrating the effectiveness of KIST-G1 as a potent anti-glioblastoma agent. Caspase-3 stain further showed the elevated apoptotic cell population in KIST-G1-treated tumor tissues (24.8 ± 1.9%) compared to PBS-treated tumor tissues (1.4 ± 0.3%) (Figure S5).

Due to the existence of the skull, the size of the brain did not increase despite the massive tumor growth in the brain (Figure 5E). Instead, shrinkage of the contralateral hemisphere was observed in the glioblastoma brain. In PBS-injected control, the contralateral brain area shrunk to 64.5 ± 9.3% of normal brain area (Figure 5F). In contrast, the KIST-G1 treated group showed no significant change in total brain size and the left brain area. Again, TMZ-treated group showed only moderate effects. As the result of brain shrinkage, significantly reduced cortical thickness and massive neuronal loss were observed in PBS-treated group (Figure 5G,H). In comparison, the KIST-G1-treated group showed almost normal cortical thicknesses and number of neurons. These results indicate that KIST-G1 was not only effective in suppressing tumor growth in the brain but also in preventing brain edema and neuronal death caused by glioblastoma.

**Figure 6:**
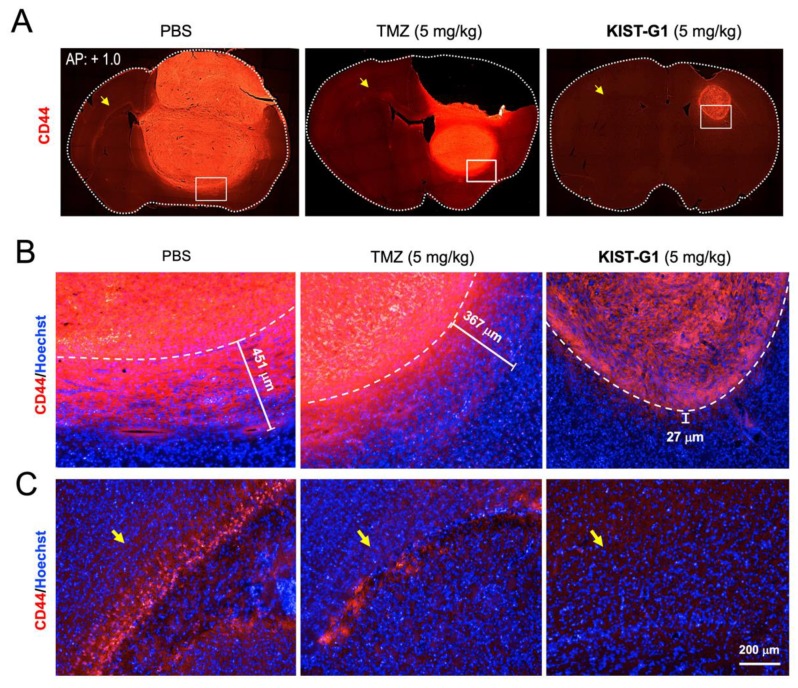
Inhibition of glioblastoma cell infiltration by KIST-G1 in orthotopic xenograft mice brain. (**A**) CD44-stains of brain tissues of PBS-, TMZ-, or KIST-G1-treated mice (AP: +1.0). (**B**) A magnification of the striatum with a representative part of the tumor margin [white square in (A)]. (**C**) A magnification of the corpus callosum in the contralateral hemisphere [yellow arrow in (A)]. U87MG cells in the contralateral hemisphere of the brain detected by anti-CD44 antibody. Nuclei were counter stained with Hoechst. Scale bar, 200 μm.

To evaluate glioblastoma cell infiltration, tumor tissues were stained with an anti-CD44 antibody. As a cell-surface marker for malignant tumors, CD44 indicates tumor cell invasion and metastasis [33]. Similar to the cresyl violet stain, the CD44 stain illuminated the dramatic antineoplastic effects in the KIST-G1-treated group (Figure 6A). The KIST-G1-treated tumor exhibited better defined tumor margins and fewer invasive cells in striatum adjacent to the glioblastoma (Figure 6B). The marginal thickness of the tumor in the PBS-treated group was 442.7 ± 68.1 μm. In contrast, the marginal thickness in the KIST-G1-treated group was merely 27.0 ± 5.7 μm, indicating the strong suppression of glioblastoma cell infiltration. Interestingly, the marginal thickness of the tumor in the TMZ-treated group was 311.0 ± 51.6 μm, which was relatively thicker compared to the moderate size of the tumor observed in the PBS-treated group. These results suggest that TMZ might be less effective in inhibiting tumor cell filtration than inhibiting tumor cell proliferation, as shown in Figure 1C. Figure 6C further shows glioblastoma cell migration to the contralateral brain region, indicated with a yellow arrow in Figure 6A. In the PBS-treated group, a number of CD44-positive glioblastoma cells were observed in the corpus callosum of the contralateral hemisphere. In both the PBS-treated and the TMZ-treated groups, a number of CD44-positive glioblastoma cells were observed on the corpus callosum in the contralateral hemisphere, indicated with a yellow arrow, whereas the KIST-G1-treated brain showed no trace of CD44-positive cells in the contralateral brain region (Figure 6C). Our results clearly indicate that KIST-G1 suppressed the glioblastoma cell migration as well as its growth in the brain. 

## 4. Discussion

Glioblastoma is one of the most deadly forms of brain tumor. Great efforts have been made to develop effective drugs preventing glioblastoma growth and migration. However, the success rate is low due to the little brain uptake of conventional anti-cancer agents. In this study, we proposed that the introduction of an aryloxazole moiety that inhibits P-gp would be beneficial in increasing the brain uptake of drug substances and demonstrated that an anti-microtubule agent bearing an aryloxazole moiety efficiently targeted glioblastoma aided by the dramatically increased BBB permeable property. It is interesting to note that KIST-G1 showed a much stronger anti-glioblastoma activity compared to TMZ, a gold standard drug for glioblastoma. KIST-G1 almost completely prevented glioblastoma cell migration and infiltration in the brain, while TMZ showed only moderate effects.

Although KIST-G1 showed superior anti-glioblastoma activity compared to TMZ, the toxicity should be carefully evaluated. Especially in the brain, microtubules play critical roles in neuronal structures and functions [34,35]. Treatment of anti-microtubule agents to the brain could cause unwanted side effects. Also, recently, colchicine-induced hepatotoxicity has been observed in cases of overdose [36]. The liver toxicity of KIST-G1, which is a derivative of colchicine, should be carefully evaluated further.

## 5. Conclusions

In this study, we demonstrated that KIST-G1 inhibited the glioblastoma cell proliferation and migration in vitro and in vivo. As a colchicine derivative containing an aryloxazole moiety, which is known to inhibit P-glycoprotein, KIST-G1 showed the higher BBB penetration property in PAMPA. Aided by the dramatically increased BBB permeable property, KIST-G1 strongly suppressed glioblastoma cell growth and almost completely prevented glioblastoma cell infiltration in the brain. All these results prove that KIST-G1 serves as a potent anti-glioblastoma agent by inhibiting glioblastoma cell growth and migration.

## Supplementary Materials

The following are available online at https://www.mdpi.com/1999-4923/11/10/497/s1, Figure S1. Anti-proliferative activity of KIST-G1; Figure S2. Liver morphology of GBM xenograft models; Figure S3. Quantification of body weight and blood creatinine; Figure S4. Western blot analysis of apoptotic markers; Figure S5. Caspase-3 analysis in GBM xenograft brain tissues; Table S1. Anti-proliferation effects (IC50) of aryloxazole derivatives; Table S2. PAMPA permeability assay; Table S3. P-glycoprotein inhibition assay; Scheme S1. Synthesis of aryloxazole derivatives (KIST-G1–G7); Note S1. Synthesis of aryloxazole derivatives; Note S2. NMR, HR-MS and HPLC characterization; Note S3. P-glycoprotein inhibition assay; Note S4. PAMPA permeability assay; Note S5. Molecular Modeling; Note S6. Docking study; Note S7. Immunoblot analysis; Note S8. Creatinine assay.

## References

[1] Louis D.N. (2006). Molecular pathology of malignant gliomas. Annu. Rev. Pathol. Mech. Dis..

[2] Reardon D.A., Rich J.N., Friedman H.S., Bigner D.D. (2006). Recent advances in the treatment of malignant astrocytoma. J. Clin. Oncol..

[3] Kleihues P., Sobin L.H. (2000). World health organization classification of tumors. Cancer.

[4] Wong E.T., Hess K.R., Gleason M.J., Jaeckle K.A., Kyritsis A.P., Prados M.D., Levin V.A., Yung W.A. (1999). Outcomes and prognostic factors in recurrent glioma patients enrolled onto phase ii clinical trials. J. Clin. Oncol..

[5] Juliano R.L., Ling V. (1976). A surface glycoprotein modulating drug permeability in chinese hamster ovary cell mutants. BBA Biomembr..

[6] Gottesman M.M., Fojo T., Bates S.E. (2002). Multidrug resistance in cancer: Role of atp–dependent transporters. Nat. Rev. Cancer.

[7] Kemper E.M., van Zandbergen A.E., Cleypool C., Mos H.A., Boogerd W., Beijnen J.H., van Tellingen O. (2003). Increased penetration of paclitaxel into the brain by inhibition of p-glycoprotein. Clin. Cancer Res..

[8] Silva R., Carmo H., Vilas-Boas V., Barbosa D.J., Palmeira A., Sousa E., Carvalho F., de Lourdes Bastos M., Remião F. (2014). Colchicine effect on p-glycoprotein expression and activity: In silico and in vitro studies. Chem. Biol. Interact..

[9] Zinzi L., Capparelli E., Cantore M., Contino M., Leopoldo M., Colabufo N.A. (2014). Small and innovative molecules as new strategy to revert mdr. Front. Oncol..

[10] Jordan M. (2002). Mechanism of action of antitumor drugs that interact with microtubules and tubulin. Curr. Med. Chem. Anti-Cancer Agents.

[11] Drion N., Lemaire M., Lefauconnier J.M., Scherrmann J.M. (1996). Role of p-glycoprotein in the blood-brain transport of colchicine and vinblastine. J. Neurochem..

[12] Colabufo N.A., Berardi F., Perrone M.G., Cantore M., Contino M., Inglese C., Niso M., Perrone R. (2009). Multi-drug-resistance-reverting agents: 2-aryloxazole and 2-arylthiazole derivatives as potent bcrp or mrp1 inhibitors. ChemMedChem.

[13] Choi M.J., No E.S., Thorat D.A., Jang J.W., Yang H., Lee J., Choo H., Kim S.J., Lee C.S., Ko S.Y. (2013). Synthesis and biological evaluation of aryloxazole derivatives as antimitotic and vascular-disrupting agents for cancer therapy. J. Med. Chem..

[14] Jiang P., Mukthavavam R., Chao Y., Bharati I.S., Fogal V., Pastorino S., Cong X., Nomura N., Gallagher M., Abbasi T. (2014). Novel anti-glioblastoma agents and therapeutic combinations identified from a collection of fda approved drugs. J. Transl. Med..

[15] Xia W., Fu W., Cai X., Wang M., Chen H., Xing W., Wang Y., Zou M., Xu T., Xu D. (2015). Angiogenin promotes u87mg cell proliferation by activating nf-κb signaling pathway and downregulating its binding partner fhl3. PLoS ONE.

[16] Koo H.-J., Shin S., Choi J.Y., Lee K.-H., Kim B.-T., Choe Y.S. (2015). Introduction of methyl groups at c2 and c6 positions enhances the antiangiogenesis activity of curcumin. Sci. Rep..

[17] Zhang Y., Zhang G.L., Sun X., Cao K.X., Ma C., Nan N., Yang G.W., Yu M.W., Wang X.M. (2018). Establishment of a murine breast tumor model by subcutaneous or orthotopic implantation. Oncol. Lett..

[18] Woo S.R., Ham Y., Kang W., Yang H., Kim S., Jin J., Joo K.M., Nam D.-H. (2014). KML001, a telomere-targeting drug, sensitizes glioblastoma cells to temozolomide chemotherapy and radiotherapy through DNA damage and apoptosis. BioMed. Res. Int..

[19] López-Valero I., Saiz-Ladera C., Torres S., Hernández-Tiedra S., García-Taboada E., Rodríguez-Fornés F., Barba M., Dávila D., Salvador-Tormo N., Guzmán M. (2018). Targeting glioma initiating cells with a combined therapy of cannabinoids and temozolomide. Biochem. Pharmacol..

[20] Salphati L., Alicke B., Heffron T.P., Shahidi-Latham S., Nishimura M., Cao T., Carano R.A., Cheong J., Greve J., Koeppen H. (2016). Brain distribution and efficacy of the brain penetrant pi3k inhibitor gdc-0084 in orthotopic mouse models of human glioblastoma. Drug Metab. Dispos..

[21] Clark M.J., Homer N., O’Connor B.D., Chen Z., Eskin A., Lee H., Merriman B., Nelson S.F. (2010). U87mg decoded: The genomic sequence of a cytogenetically aberrant human cancer cell line. PLoS Genet..

[22] Roos W., Batista L., Naumann S., Wick W., Weller M., Menck C., Kaina B. (2007). Apoptosis in malignant glioma cells triggered by the temozolomide-induced DNA lesion *O*^6^-methylguanine. Oncogene.

[23] Avdeef A., Strafford M., Block E., Balogh M.P., Chambliss W., Khan I. (2001). Drug absorption in vitro model: Filter-immobilized artificial membranes: 2. Studies of the permeability properties of lactones in piper methysticum forst. Eur. J. Pharm. Sci..

[24] Di L., Kerns E.H., Fan K., McConnell O.J., Carter G.T. (2003). High throughput artificial membrane permeability assay for blood–brain barrier. Eur. J. Med. Chem..

[25] Nicklisch S.C., Rees S.D., McGrath A.P., Gökirmak T., Bonito L.T., Vermeer L.M., Cregger C., Loewen G., Sandin S., Chang G. (2016). Global marine pollutants inhibit p-glycoprotein: Environmental levels, inhibitory effects, and cocrystal structure. Sci. Adv..

[26] Bansal T., Mishra G., Jaggi M., Khar R.K., Talegaonkar S. (2009). Effect of p-glycoprotein inhibitor, verapamil, on oral bioavailability and pharmacokinetics of irinotecan in rats. Eur. J. Pharm. Sci..

[27] Loo T.W., Clarke D.M. (1999). Identification of residues in the drug-binding domain of human p-glycoprotein analysis of transmembrane segment 11 by cysteine-scanning mutagenesis and inhibition by dibromobimane. J. Biol. Chem..

[28] Fan L., Yang Q., Tan J., Qiao Y., Wang Q., He J., Wu H., Zhang Y. (2015). Dual loading mir-218 mimics and temozolomide using aucooh@ fa-cs drug delivery system: Promising targeted anti-tumor drug delivery system with sequential release functions. J. Exp. Clin. Cancer Res..

[29] Kim J.T., Kim J.-S., Ko K.W., Kong D.-S., Kang C.-M., Kim M.H., Son M.J., Song H.S., Shin H.-J., Lee D.-S. (2006). Metronomic treatment of temozolomide inhibits tumor cell growth through reduction of angiogenesis and augmentation of apoptosis in orthotopic models of gliomas. Oncol. Rep..

[30] Cen L., Carlson B.L., Pokorny J.L., Mladek A.C., Grogan P.T., Schroeder M.A., Decker P.A., Anderson S.K., Giannini C., Wu W. (2013). Efficacy of protracted temozolomide dosing is limited in mgmt unmethylated gbm xenograft models. Neuro-Oncology.

[31] Kluck R.M., Bossy-Wetzel E., Green D.R., Newmeyer D.D. (1997). The release of cytochrome c from mitochondria: A primary site for Bcl-2 regulation of apoptosis. Science.

[32] Soldani C., Scovassi A. (2002). Poly (ADP-ribose) polymerase-1 cleavage during apoptosis: An update. Apoptosis.

[33] Merzak A., Koocheckpour S., Pilkington G.J. (1994). Cd44 mediates human glioma cell adhesion and invasion in vitro. Cancer Res..

[34] Dent E.W., Baas P.W. (2014). Microtubules in neurons as information carriers. J. Neurochem..

[35] Kapitein L.C., Hoogenraad C.C. (2015). Building the neuronal microtubule cytoskeleton. Neuron.

[36] Abbott C.E., Xu R., Sigal S.H. (2017). Colchicine-induced hepatotoxicity. ACG Case Rep. J..

