# Supplementary material for: Development of an Aryloxazole Derivative as a Brain-Permeable Anti-Glioblastoma Agent
Source: Pharmaceutics. 2019 Sep 28;11(10):497. doi: 10.3390/pharmaceutics11100497 (PMC6835410; doi:10.3390/pharmaceutics11100497)
Supplement: Supplementary file 1 [file pharmaceutics-11-00497-s001.pdf]

pharmaceutics

MDPI

Supplementary Materials: Development of an Aryloxazole Derivative as a Brain-Permeable Anti-Glioblastoma Agent

Seulgi Shin, Sungsu Lim, Ji Yeon Song, Dohee Kim, Min Jeong Choi, Changdev G. Gadhe, A Young Park, Ae Nim Pae and Yun Kyung Kim

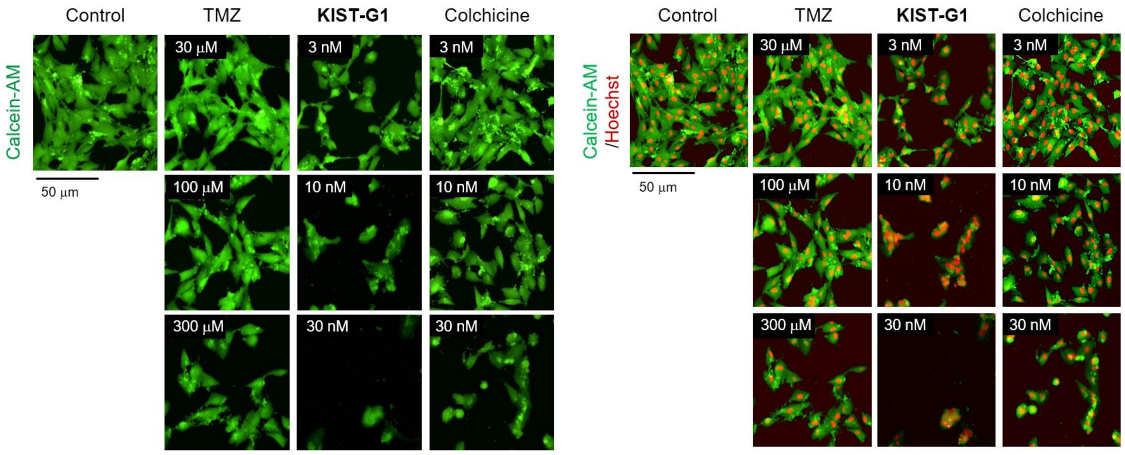

Figure S1. Anti-proliferative activity of KIST-G1. U87MG cells were treated with TMZ, KIST-G1, or colchicine at diverse concentrations. After 48 h, cells were stained with calcein-AM (green) and Hoechst (red) to determine cell viability and proliferation. Scale bar, 50 μm.

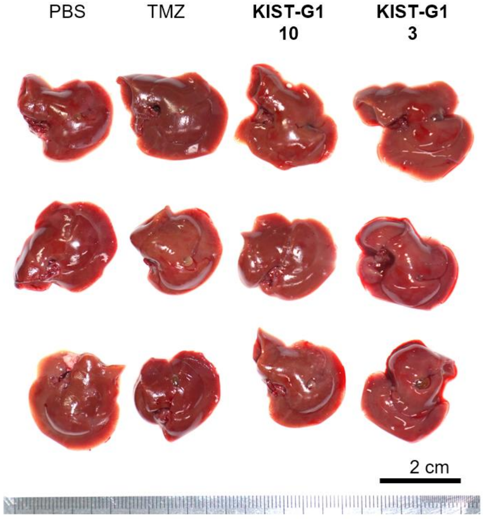

Figure S2. Liver morphology of GBM xenograft models. Macroscopic image of livers from PBS-, TMZ-, or KIST-G1 (3 mg/kg or 10 mg/kg)-treated subcutaneous U87MG-xenograft mice. Scale bar, 2 cm.

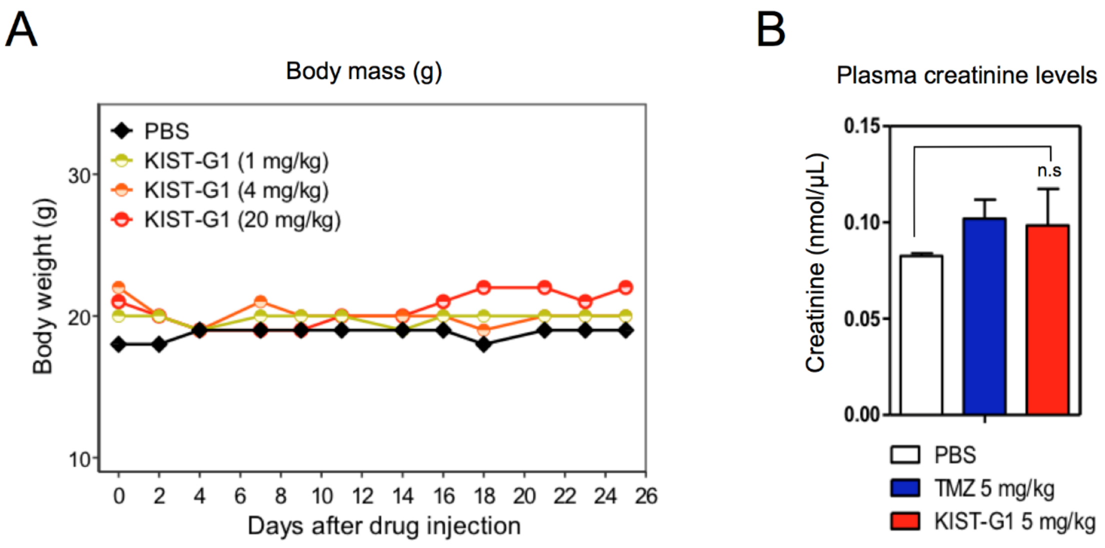

Figure S3. Quantification of body weight and blood creatinine. (**A**,**B**) To evaluate drug-induced toxicity, body weights and blood creatinine levels were measured after the repeated injection of PBS, TMZ, or KIST-G1 to C57BL/6 mice for 28 days. (**A**) Body weights were measured every other day during PBS, or KIST-G1 (1 mg/kg, 4 mg/kg, or 20 mg/kg) administration. Data were represented as means ± SEM (n = 3/group). (**B**) After the repeated injection of PBS, TMZ (5 mg/kg), or KIST-G1 (5 mg/kg) for 28 days, blood creatinine levels were quantified. In all groups, creatinine levels were normal, ranging 0.08 to 0.11 mg/dL levels [1]. Error bars indicate standard deviations from four animals in each group, * $p < 0.05$.

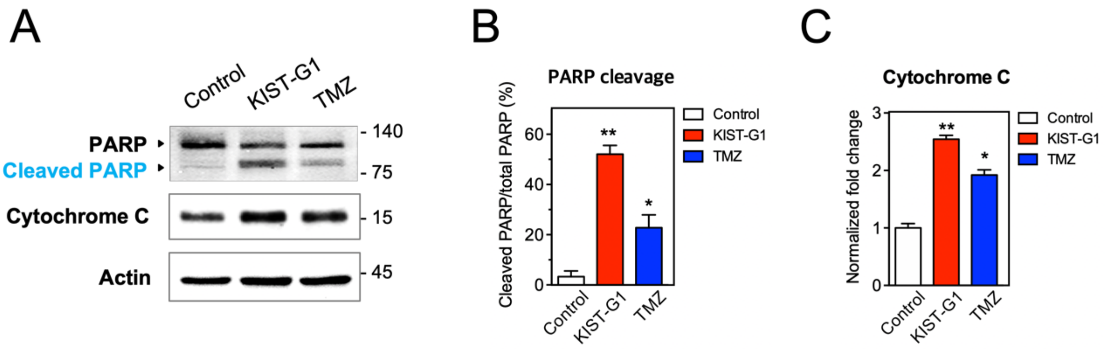

Figure S4. Western blot analysis of apoptotic markers. (**A**) U87MG cells were treated with KIST-G1 (3 nM), or TMZ (300 μM) for 24 h. Cytochrome C expression and PARP cleavage were evaluated by using anti-PARP and anti-cytochrome C antibodies. Anti-β-actin antibody was used for loading control. (**B**) Quantification of PARP cleavage and cytochrome C expression. Each bar represents the mean ± SD calculated from three independent experiments, * $p < 0.05$ and ** $p < 0.01$ compared with control.

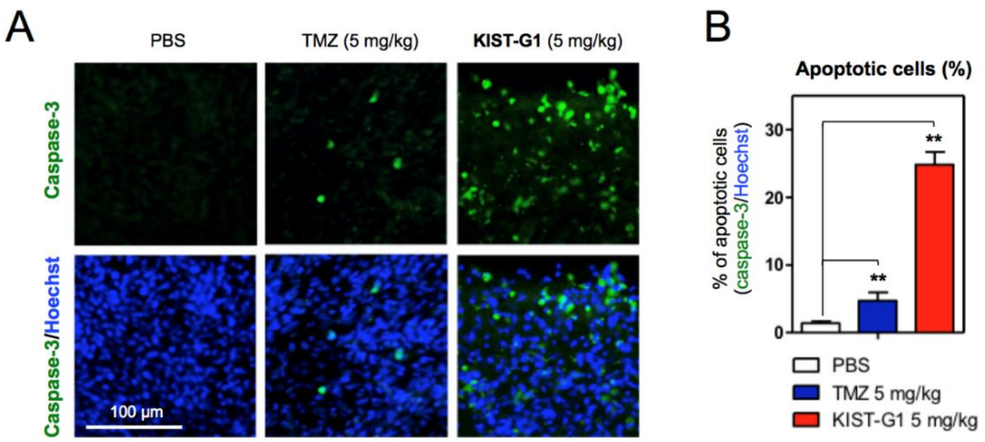

Figure S5. Caspase-3 analysis in GBM xenograft brain tissues. (**A**) Immuno-fluorescent images with anti-cleaved caspase-3 (Green). Nuclei were counter-stained with Hoechst (Blue). Scale bar, 100 μm. (**B**) Percentage of apoptotic cells in histological tumor regions of U87MG orthotopic xenografts. Data was expressed as the mean and the standard error of the mean, $n = 4$, ** $p < 0.01$.

Table S1. Anti-proliferation effects (IC_{50}) of aryloxazole derivatives.

**IC_{50} (nM)**	**A172**	**U251**	**U373**	**T98G**	**HS683**	**U87MG**
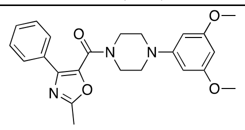 **KIST-G1**	8	4	10	3	12	3
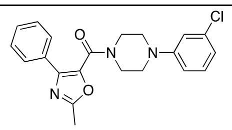 **KIST-G2**	48	30	121	45	64	46
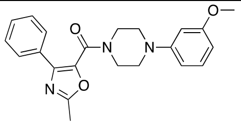 **KIST-G3**	22	72	38	33	37	34
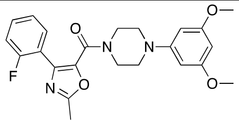 **KIST-G4**	24	10	41	16	42	33
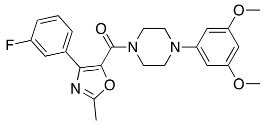 **KIST-G5**	240	466	419	211	383	316
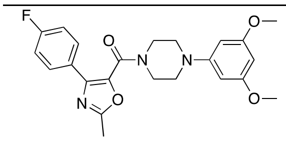 **KIST-G6**	36	132	45	32	36	42
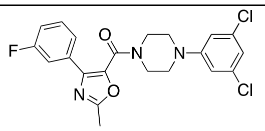 **KIST-G7**	215	308	572	158	1,938	208
**Colchicine**	16	13	14	33	33	13
**Temozolomide**	256,743	451,380	109,138	473,631	121,468	392,267

Table S2. PAMPA permeability assay.

Test	P_e (10^{-6} cm/s)				
	1st	2nd	3rd	Avg	STDEV
**Progesterone**	40.57	40.65	44.40	41.87	2.19
**Lidocaine**	23.25	23.08	22.40	22.91	0.45
**Theophylline**	0.24	0.26	0.25	0.25	0.01
**Colchicine**	0.82	0.79	0.82	0.81	0.02
**KIST-G1**	52.04	51.15	51.81	51.67	0.46

Table S3. P-glycoprotein inhibition assay.

Test	Direction	P_{app} (10^{-5} cm/s)			Avg	STDEV
		1st	2nd	3rd		
**DMSO**	A to B	0.58	0.57	0.58	0.57	0.01
	B to A	4.26	5.35	6.02	5.21	0.89
**Verapamil**	A to B	4.38	3.98	4.45	4.27	0.26
	B to A	3.87	3.47	4.46	3.93	0.50
**Colchicine**	A to B	0.60	0.59	0.60	0.60	0.01
	B to A	6.56	6.40	6.46	6.47	0.08
**KIST-G1**	A to B	1.00	1.05	1.07	1.04	0.03
	B to A	5.74	5.28	6.00	5.67	0.37

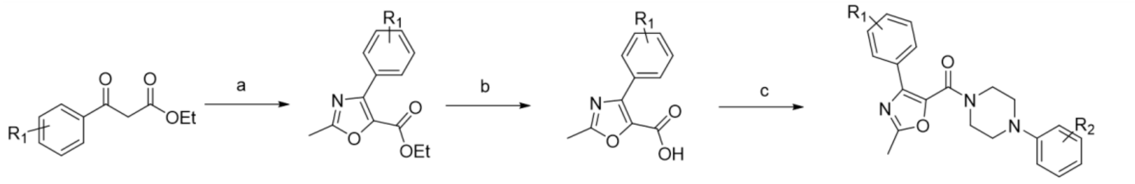

1 2 3 4

KIST-G1: R_1=H, R_2=3,5-di-OCH3
KIST-G2: R_1=H, R_2=Cl
KIST-G3: R_1=H, R_2=3-OCH3
KIST-G4: R_1=2-F, R_2=3,5-di-OCH3
KIST-G5: R_1=3-F, R_2=3,5-di-OCH3
KIST-G6: R_1=4-F, R_2=3,5-di-OCH3
KIST-G7: R_1=3-F, R_2=3,5-di-Cl

Scheme S1. Synthesis of aryloxazole derivatives (KIST-G1–G7). Reagents and conditions: (a) (i) HDNIB, CH_3CN, reflux, 1 h; (ii) acetamide (or thioacetamide), microwave 80 W, 1 min; (b) (i) 1 N NaOH; (ii) 1 N HCl; (c) arylpiperazine, EDCI, HOBt, NMM, CH_2Cl_2.

Note S1. Synthesis of aryloxazole derivatives

Synthetic methods of aryloxazole derivatives in Table S1 were described in our previous study [2]. The oxazole core scaffold **2** was synthesized from commercially available ethyl 3-oxo-3-phenyl-propanoate in the presence of HDNIB ([hydroxy(2,4-dinitro benzenesulfonyloxy)iodo]benzene) in one pot. The reaction mixture first formed an α-sulfonyloxyl ketone as the intermediate and then the treatment of acetamide in combination with a microwave irradiation at 80 W for 1 min, the heterocyclic core **4** was obtained over 20 % yield. Hydrolysis of **2** afforded the acid fragment **3** in 80% yield. The amide coupling reaction was carried out with various aryl piperazines by using EDCI (1-Ethyl-3-(3-dimethylaminopropyl)carbodiimide) to obtain the desired aryloxazole derivatives **(KIST-G1–G7)** over 60 % yield (**Scheme S1**).

Note S2. NMR, HR-MS and HPLC characterization

(4-(3,5-Dimethoxyphenyl)piperazin-1-yl)(2-methyl-4-phenyloxazol-5-yl)methanone (KIST-G1)

^{1}H NMR (300 MHz, $CDCl_3$) δ 7.75–7.72 (dd, *J* = 8.22 Hz, 1.62 Hz, 2H), 7.45–7.36 (m, 3H), 6.05–6.01 (m, 3H), 3.91 (br, 2H), 3.76 (s, 6H), 3.42 (br, 2H), 3.23 (br, 2H), 2.89 (br, 2H), 2.17 (s, 3H); ^{13}C NMR (75 MHz, $CDCl_3$) δ 161.67, 161.52, 159.85, 152.68, 140.40, 137.76, 130.40, 129.09, 128.77, 127.61, 95.68, 92.35, 55.26, 49.42, 30.91, 14.04; mp 121–123 °C; HPLC purity 100%; HR-MS *m/z* [M + H]$^+$ (ESI$^+$) calcd. for $C_{23}H_{26}N_3O_4$ = 408.1918, found 408.1935.

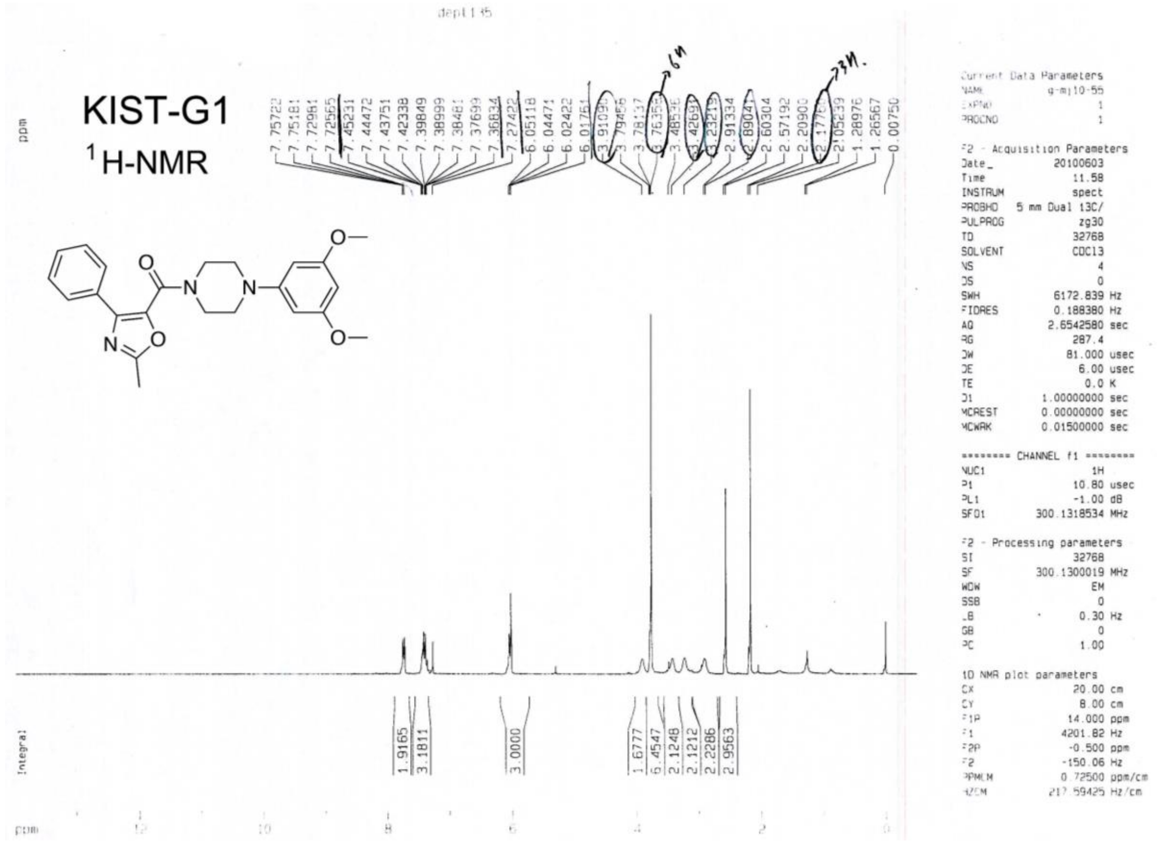

^{1}H-NMR spectrum of KIST-G1.

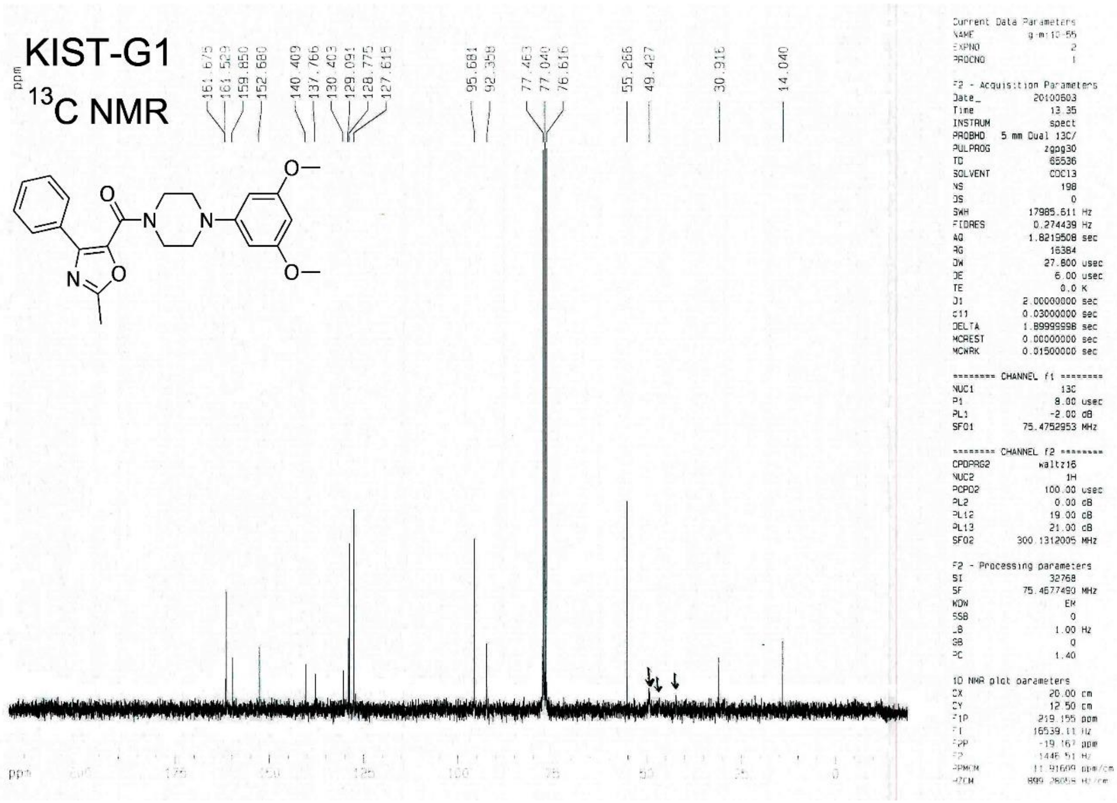

^{13}C-NMR spectrum of KIST-G1.

(4-(3-Chlorophenyl)piperazin-1-yl)(2-methyl-4-phenyloxazol-5-yl)methanone (KIST-G2)

^{1}H NMR (400 MHz, $CDCl_3$) δ 7.75–7.73 (m, 2H), 7.41–7.36 (m, 3H), 7.15–7.13(m, 1H), 6.85–6.83 (m, 1H), 6.81(s, 1H), 6.80(d, *J* = 2.00 Hz, 1H), 3.89 (br, 2H), 3.46 (br, 2H), 3.22 (br, 2H), 2.91 (br, 2H), 2.59 (s, 3H); ^{13}C NMR (100 MHz, $CDCl_3$) *δ* 161.74, 159.86, 151.74, 140.58, 137.69, 135.05, 130.38, 130.20, 129.16, 128.80, 127.65, 120.33, 116.48, 114.52, 50.22, 48.80, 46.62, 42.13, 14.07; mp 191–193 °C; HPLC purity 99%; HR-MS *m/z* $[M + H]^+$ (ESI^+) calcd. for $C_{21}H_{21}ClN_3O_2$ = 382.1317, found 382.1328.

(4-(3-Methoxyphenyl)piperazin-1-yl)(2-methyl-4-phenyloxazol-5-yl)methanone (KIST-G3)

^{1}H NMR (300 MHz, $CDCl_3$) *δ* 7.77–7.73 (dd, *J* = 8.28 Hz, 1.65 Hz, 2H), 7.45–7.37 (m, 3H), 7.18 (t, *J* = 8.18 Hz, 1H), 6.50–6.45 (td, *J* = 5.94 Hz, 2.24 Hz, 2H), 6.41 (s, 1H), 3.92 (br, 2H), 3.78 (s, 3H), 3.43 (br, 2H), 3.24 (br, 2H), 2.93 (br, 2H), 2.57 (s, 3H); ^{13}C NMR (75 MHz, $CDCl_3$) *δ* 161.63, 160.64, 159.86, 152.10, 140.40, 137.80, 130.42, 129.94, 129.06, 128.75, 127.61, 109.37, 105.35, 103.25, 55.21, 49.33, 46.25, 14.02; mp 162–165 °C; HPLC purity 97%; HR-MS *m/z* $[M + H]^+$ (ESI^+) calcd. for $C_{22}H_{24}N_3O_3$ = 378.1812, found 378.1828.

(4-(3,5-Dimethoxyphenyl)piperazin-1-yl)(4-(2-fluorophenyl)-2-methyloxazol-5-yl)methanone (KIST-G4)

^{1}H NMR (300 MHz, $CDCl_3$) *δ* 7.78–7.72 (td, *J* = 1.27 Hz, 7.51 Hz, 1H), 7.39–7.33 (m, 1H), 7.27–7.21 (m, 1H), 7.15–7.09 (m, 1H), 6.05 (s, 3H), 3.79 (br, 2H), 3.77 (s, 6H), 3.53 (br, 2H), 3.21–2.96 (br, 4H), 2.58 (s, 3H); ^{13}C NMR (75 MHz, $CDCl_3$) *δ* 161.56, 161.36, 159.38, 152.77, 140.19, 134.83, 130.78 (d, *J* = 33.9 Hz), 130.30, 124.54, 118.75, 116.06, 115.78, 95.71, 92.34, 55.26, 49.30, 14.19; mp 221–222 °C; HPLC purity 95%; FABMS (m/z): $[M^+ + H]$ calcd for $C_{23}H_{25}FN_3O_4$ = 426.1751, found 426.1829; HR-MS *m/z* $[M + H]^+$ (ESI^+) calcd. for $C_{23}H_{25}FN_3O_4$ = 426.1824, found 426.1841.

(4-(3,5-Dimethoxyphenyl)piperazin-1-yl)(4-(3-fluorophenyl)-2-methyloxazol-5-yl)methanone (KIST-G5)

^{1}H NMR (300 MHz, $CDCl_3$) *δ* 7.58–7.52 (m, 2H), 7.39–7.37 (m, 1H), 7.03–7.06 (m, 1H), 6.05 (s, 3H), 3.92 (br, 2H), 3.77 (s, 6H), 3.49 (br, 2H), 3.25 (br, 2H), 3.00 (br, 2H), 2.57 (s, 3H); ^{13}C NMR (75 MHz, $CDCl_3$) *δ* 161.55, 161.49, 159.53, 152.64, 138.31, 132.22, 130.34, 123.25, 116.06, 115.77, 114.73, 114.42,

95.74, 92.44, 55.27, 49.30, 46.70, 42.30, 30.91, 13.99; mp 182–184 °C; HPLC purity 96%; HR-MS *m/z* [M + H]$^+$ (ESI$^+$) calcd. for $C_{23}H_{25}FN_3O_4$ = 426.1824, found 426.1841.

(4-(3,5-Dimethoxyphenyl)piperazin-1-yl)(4-(4-fluorophenyl)-2-methyloxazol-5-yl)methanone (KIST-G6)

^{1}H NMR (300 MHz, $CDCl_3$) δ 7.83–7.78 (m, 2H), 7.14–7.07 (m, 2H), 6.05 (s, 3H), 3.90 (br, 2H), 3.77 (s, 3H), 3.52 (br, 2H), 3.24 (br, 2H), 3.02 (br, 2H), 2.17 (s, 3H); ^{13}C NMR (75 MHz, $CDCl_3$) δ 161.58, 161.45, 159.67, 152.65, 140.08, 137.68, 129.68, 126.64, 115.86, 115.57, 95.73, 92.42, 55.26, 49.63, 48.56, 13.97; mp 171–173 °C; HPLC purity 98%; HR-MS *m/z* [M + H]$^+$ (ESI$^+$) calcd. for $C_{23}H_{25}FN_3O_4$ = 426.1824, found 426.1841.

(4-(3,5-Dichlorophenyl)piperazin-1-yl)(4-(3-fluorophenyl)-2-methyloxazol-5-yl)methanone (KIST-G7)

^{1}H NMR (300 MHz, $CDCl_3$) δ 7.59–7.53 (m, 2H), 7.42–7.35 (m, 1H), 7.11–7.04 (tt, J = 0.97 Hz, 2.51 Hz, 8.27 Hz, 1H), 6.85 (t, J = 1.57 Hz, 1H), 6.72 (d, J = 1.63 Hz, 2H), 3.91 (br, 2H), 3.49 (br, 2H), 3.27 (br, 2H), 3.04 (br, 2H), 2.57 (s, 3H); ^{13}C NMR (75 MHz, $CDCl_3$) δ 164.54, 161.55, 161.28, 159.55, 152.01, 139.85, 138.12, 135.63, 132.49 (d, J = 33.3 Hz), 130.35 (d, J = 30 Hz), 123.31, 119.95, 116.16, 115.88, 114.81, 114.46, 48.66, 47.96, 46.37, 42.05, 14.00; mp 138–139 °C; HPLC purity 96%; HR-MS *m/z* [M + H]$^+$ (ESI$^+$) calcd. for $C_{21}H_{19}Cl_2FN_3O_2$ = 434.0833, found 434.0832.

Note S3. P-glycoprotein inhibition assay

P-glycoprotein (P-gp) inhibition assay was performed according to the LLC-PK1-MDR1 monolayer efflux assay protocol as previously described [3]. For P-gp inhibition studies, LLC-PK1-MDR1 cells expressing human P-gp were seeded onto polycarbonate transwell filter membranes at a density of 1.5 × 10^5 cells/well. Both the test compound (KIST-G1, colchicine or verapamil) and P-gp substrate (quinidine) were diluted for studies in transport buffer (10 mM glucose, 4 mM sodium bicarbonate, 1 mM HEPES in HBSS (pH 7.4)). Each compound was tested at 10 μM concentration in both the apical-to-basolateral (A→B) and basolateral-to-apical (B→A) directions for 1 hr at 37 °C. Samples were first taken from both the apical and basolateral compartments every 15 min throughout the test period and then the concentration of quinidine was analyzed by a LC-MS/MS method. The permeability coefficient (P_{app}) was calculated according to the following equation: $P_{app} = dQ/dt/C_0A$. dQ/dt is the flux of the test compound across the monolayer, A is the surface area of the cell monolayer, and C_0 is the initial concentration in the donor compartment. The P_{app} values were expressed as *cm/sec*. The permeability coefficient has been reported as 'Degree of P-gp inhibition (%)' of quinidine transport by the test compounds: P-gp inhibition (%) = $\{1 - (R_i - 1)/(R - 1)\} \times 100$. R_i is the ratio of B to A permeability to A to B permeability of quinidine in the presence of the test compound. R is the ratio of B to A permeability to A to B permeability of quinidine in the absence of test compound.

Note S4. PAMPA permeability assay

PAMPA (parallel artificial membrane permeability assay) was performed according to Double-sink PAMPA with direct UV detection protocol from *pION, Inc.* as previously described [4]. PAMPA experiment was carried out in a 96-well format. A 96-well microtiter plate and a 96-well filter plate were assembled into a 'sandwich' such that each composite well was separated by a 125 μm micro-filter disc. Test compound (KIST-G1 or colchicine) and reference compound (progesterone, lidocaine or thophyline) were diluted in donor *pION* buffer, pH 7.4 at final concentrations of 50 μM. Acceptor wells at top of the sandwich were rehydrate with 200 μL of *pION* buffer, pH 7.4. The sandwich construct was then incubated at room temperature. After 4 hrs, the sandwich construct was dissembled. The solution in the acceptor wells and the donor wells were transferred to a disposable UV-transparent plate (*pION,* Inc, USA). UV absorptions were measured with a SPECTRAMax microplate spectrophotometer at absorption wavelengths between 250 and 498 nm. *pION* PAMPA Explorer Software (ver. 3.8) was used to process the data.

Note S5. Molecular Modeling

For P-gp, many X-ray crystal structures were available in protein data bank (PDB). However, for human P-gp, X-ray crystal structure remains yet to be solved. Primary sequences of human and mouse P-gp were taken from the Universal Protein Resource (http://www.uniprot.org) with the accession code P08183 and P21447, respectively. A Basic Local Alignment Search Tool (BLAST) was performed for the human P-gp sequence against PDB structures to identify suitable templates. Mouse P-gp structures were the topmost templates with a sequence identity of 87% and query coverage of 99%. Binding site residues are mostly conserved among both the human and mouse. Thus, we decided to directly use the mouse P-gp structure in this study. We used a mouse X-ray crystal structure (PDB codes 4XWK) to further model and guide a docking study. Chemical structures of colchicine and KIST-G1 were sketched using ChemDraw Professional 15.0 program and saved as SDF format. Ligand structures were imported to the maestro and converted into 3D-format using default LigPrep setting at 7.4 pH value. OPLS2005 force fields were utilized for the preparation and minimization of the ligand conformations. 4XWK was prepared using protein preparation wizard with the default setting. Authors will release the atomic coordinates and experimental data upon article publication.

Note S6. Docking study

All the docking calculations were performed using Schrodinger's GLIDE (Schrodinger 2015-1 LLC, New York, NY) module. Information available in the literature about mutagenesis [5,6] and modeling [7,8] combined with the co-crystal ligands information and a binding site for docking study was defined. Glide standard precision (SP) docking algorithm was used and fifty poses were generated for each ligand and ranked according to a more negative Gscore value. The Gscore function is comprised of terms for H-bond, hydrophobic, van-der-Waals interaction energy, and ligand strain energy. All the figures were rendered using Discovery Studio Client 2017 R2 package.

Note S7. Immunoblot analysis

For immunoblot analysis of apoptosis-associated proteins, U87MG cells grown in a 6-well plate were treated with KIST-G-1 (3 nM), or TMZ (300 μM) for 24 h. Ten micrograms of cell lysates were analyzed with anti-PARP (Cell Signaling), and anti-cytochrome C (Cell Signaling) antibodies. Anti-β-actin antibody was used for loading control. Band intensity was quantified using Image J software (NIH).

Note S8. Creatinine assay

For renal function monitoring, C57BL/6 normal mice were injected intraperitoneally with PBS, TMZ (5 mg/kg), or KIST-G1 (5 mg/kg) every other day for a period of 25 days. On the day of the sacrifice, blood was collected immediately, and serum levels of creatinine were measured with a creatinine colorimetric assay kit (Sigma-Aldrich, St. Louis, MO).

References

1. Brosius, F.C., 3rd; Alpers, C.E.; Bottinger, E.P.; Breyer, M.D.; Coffman, T.M.; Gurley, S.B.; Harris, R.C.; Kakoki, M.; Kretzler, M.; Leiter, E.H., et al. Mouse models of diabetic nephropathy. *J Am Soc Nephrol* **2009**, *20*, 2503–2512, doi:10.1681/ASN.2009070721.
2. Choi, M.J.; No, E.S.; Thorat, D.A.; Jang, J.W.; Yang, H.; Lee, J.; Choo, H.; Kim, S.J.; Lee, C.S.; Ko, S.Y. Synthesis and biological evaluation of aryloxazole derivatives as antimitotic and vascular-disrupting agents for cancer therapy. *Journal of medicinal chemistry* **2013**, *56*, 9008–9018.
3. Bansal, T.; Mishra, G.; Jaggi, M.; Khar, R.K.; Talegaonkar, S. Effect of P-glycoprotein inhibitor, verapamil, on oral bioavailability and pharmacokinetics of irinotecan in rats. *European Journal of Pharmaceutical Sciences* **2009**, *36*, 580–590, doi:https://doi.org/10.1016/j.ejps.2008.12.005.

4. Di, L.; Kerns, E.H.; Fan, K.; McConnell, O.J.; Carter, G.T. High throughput artificial membrane permeability assay for blood–brain barrier. *European Journal of Medicinal Chemistry* **2003**, *38*, 223–232, doi:https://doi.org/10.1016/S0223-5234(03)00012-6.
5. Loo, T.W.; Clarke, D.M. Identification of residues in the drug-binding domain of human P-glycoprotein. Analysis of transmembrane segment 11 by cysteine-scanning mutagenesis and inhibition by dibromobimane. *J Biol Chem* **1999**, *274*, 35388–35392.
6. Loo, T.W.; Clarke, D.M. Identification of residues within the drug-binding domain of the human multidrug resistance P-glycoprotein by cysteine-scanning mutagenesis and reaction with dibromobimane. *J Biol Chem* **2000**, *275*, 39272–39278, doi:10.1074/jbc.M007741200.
7. Ferreira, R.J.; Ferreira, M.-J.U.; dos Santos, D.J.V.A. Molecular Docking Characterizes Substrate-Binding Sites and Efflux Modulation Mechanisms within P-Glycoprotein. *Journal of Chemical Information and Modeling* **2013**, *53*, 1747–1760, doi:10.1021/ci400195v.
8. Martin, C.; Berridge, G.; Higgins, C.F.; Mistry, P.; Charlton, P.; Callaghan, R. Communication between Multiple Drug Binding Sites on P-glycoprotein. *Molecular Pharmacology* **2000**, *58*, 624.
